# Dynamic Image Analysis To Evaluate Subvisible Particles During Continuous Drug Infusion In a Neonatal Intensive Care Unit

**DOI:** 10.1038/s41598-017-10073-y

**Published:** 2017-08-24

**Authors:** Maxime Perez, Bertrand Décaudin, Aurélie Maiguy-Foinard, Christine Barthélémy, Gilles Lebuffe, Laurent Storme, Pascal Odou

**Affiliations:** 1 Univ. Lille, CHU Lille, EA 7365 – GRITA – Groupe de Recherche sur les Formes Injectables et les Technologies Associées, F-59000 Lille, France; 2 CHU Lille, Pharmacie, F-59000 Lille, France; 3 CHU Lille, Clinique d’Anesthésie-Réanimation, F-59000 Lille, France; 4 CHU Lille, Clinique de Néonatalogie, F-59000 Lille, France; 5 Univ. Lille, CHU Lille, EA 4489 – Environnement Périnatal et Santé, F-59000 Lille, France

## Abstract

Studies have shown that infused particles lead to numerous complications such as inflammation or organ dysfunctions in critically ill children. Nevertheless, there is very little data available to evaluate the amount of particulate matter potentially administered to patients, and none with regard to infants. We have investigated the quantity received by these patients during multidrug IV therapies. Two different protocols commonly used in our neonatal intensive care unit (NICU) to manage excessively preterm infants were reproduced in the laboratory and directly connected to a dynamic particle analyser. The particulate matter of infused therapies was measured over 24 h, so that both overall particulate contamination and particle sizes could be determined. No visible particles were observed during drug infusions. Particulate analyses showed a significant number of particles that can reach 85,000 per day, with peaks during discontinuous drug infusions. Moreover, we showed that very large particles of about 60 µm were infused to infants. This study showed that despite very low infusion flow rates, infants may receive a large number of particles during drug infusion, especially in NICUs. Particulate contamination of IV fluids is not without consequences for fragile infants. Preventive solutions could be effective, such as the use of in-line filters.

## Introduction

The United States Pharmacopoeia (USP)^1^ and the European Pharmacopoeia (EP)^2^, define particulate matter observed during intravenous (IV) infusions as undissolved particles from infused solutions. All injectable drugs contain particulate matter and this contamination of IV solutions is considered to be hazardous. Healthcare providers should be aware of the problem^3^.

The physico-chemical properties of injectable particles (i.e. number, size, shape and composition) have clinical consequences for patients. Several studies have shown that lungs are particularly affected by the IV administration of particles. Pulmonary capillaries represent a significant barrier to particles by their mean diameter (approximately 2 to 15 µm)^4^. Niehaus *et al*. demonstrated that around one third of injected particles are trapped in the lungs^5^ and those larger than 300 µm tend to locate there primarily^6^. Smaller particles may pass through the pulmonary capillary bed and diffuse into various extrapulmonary organs, such as the liver and spleen, before being eliminated by the reticuloendothelial system^7^. These microparticles may have deleterious effects on organ functions. Recent studies conducted in pediatric intensive care units (ICUs) have demonstrated that particle infusion causes several complications (respiratory, renal or hematologic), systemic inflammations^8–11^ and/or granuloma^12^. The risk of particle generation and their related adverse effects is higher in the newborn population, especially in preterm babies. The most immature preterm infants require prolonged parenteral nutrition to sustain growth as well as multiple drug infusions. Because of limited venous accesses, all IV solutions are routinely infused through one single catheter. The rates of infusion are extremely low, extending contact time between the various components of the mixture. Moreover, right to left shunting through the foramen ovale and/or the ductus arteriosus is frequently observed in preterm infants, especially in those with persistent pulmonary hypertension^13^. In such conditions, the particles are mainly oriented towards extrapulmonary tissue and the brain, which may cause tissue and brain damage.

Particles may occur as a result of precipitate, due to physical and/or chemical drug incompatibilities. Many studies exist on the critical problem of drug incompatibilities in neonatology^14, 15^. Pediatric and neonatal ICU patients receive many injectable drugs simultaneously and even if administered separately, they may generate particles leading to complications or death^16^. A study has indeed reported the death of neonates and infants after receiving an association of calcium and ceftriaxone^17^. Administration of low drug volumes in neonates and children can lead to an incomplete dissolution of drugs and increase the risk of particle formation. Precipitates are usually considered as physical and visible phenomena, but we demonstrated in a previous study that most infused particulate matter is sub-visible in pediatric multi-infusion IV therapy^18^. The detection of visible particles is a very subjective concept depending on the operator who performs the analysis. Melchore showed that the probability of detecting 50 µm particles is close to zero^19^. The human eye can detect a 100 and 200 µm particle in approximately 40% and ≥95% of cases, respectively. This detection limitation complicates the control of particulate contamination during IV infusions.

The aim of our study was to evaluate the true particulate contamination potentially received by infants during the IV infusion of two different multidrug protocols in our neonatal intensive care unit (NICU). The study proposes a new method to assess particulate matter using an innovative approach with a dynamic particle counter.

## Results

### Data acquisition

A mean delay of 6.25 seconds [5.94 sec; 6.51 sec] was necessary for the particles in a tricalcium phosphate suspension to be detectable by camera when the suspension was infused at neonate flow rates. Thus, during the infusion period (i.e. 24 hours), particle analysis time was set at 1 second and particle analysis was repeated every 6 seconds.

### Particle size

#### In extemporaneously prepared BPN bags at the start of infusion by static count (APSS-2000 instrument)

All solutions for infusion were supplied in containers with a nominal content <100 mL. Table 1 shows that the particulate matter of each BPN component is in accordance with the specifications of the USP and EP, for particle sizes ≥10 and 25 µm. The overall particulate matter of neonatal BPN bags was 341.1 [309.2; 358.4] and 387.4 [315.2; 402.2] particles with a size range ≥10 µm for protocols 1 and 2, respectively. However, the rate of particles ≥25 µm was below the maximum standards of the Pharmacopeia, for either neonate protocol. When adding new drugs in protocol 2, only sodium heparin and hydrocortisone hemisuccinate presented high particulate contamination for particles ≥10 µm (i.e. 32.0 [31.2; 36.1] and 61.4 [59.0; 63.2] particles, respectively).

**Table 1:** Conventional formulation of parenteral nutrition bags prepared in our NICU for our study and associated with continuous and discontinuous infused drugs. Particulate matter is expressed as mean ± standard deviations.

Component	Protocol 1	Protocol 2	Particulate matter (number of particles per mL for sizes ≥10 µm; ≥25 µm)* in each component
**Conventional formulation**			
50% dextrose^a^	3 mL	15 mL	8.1 ± 3.5; 0.5 ± 0.3
Amino acids^b^	22 mL	30 mL	30.2 ± 3.9; 0.9 ± 0.8
Water for injection^c^	17 mL	37 mL	7.2 ± 3.6; 0.4 ± 0.3
Calcium gluconate^d^	3.6 mL	4.5 mL	8.9 ± 0.3; 0.1 ± 0.1
Potassium chloride^d^	0.3 mL	0.7 mL	22.9 ± 4.2; 1.0 ± 0.3
Magnesium chloride^e^	0.5 mL	0.6 mL	17.8 ± 1.8; 0.8 ± 0.4
Glucose-1-phosphate^f^	0.8 mL	2.5 mL	20.0 ± 7.6; 0.4 ± 0.7
Trace elements^g^	0.6 mL	0.7 mL	48.8 ± 14.3; 0.8 ± 0.4
L-carnitine^h^	0.1 mL	0.1 mL	7.2 ± 0.5; 0.4 ± 0.1
Total volume	48.1 mL	91.3 mL	
**Continuous and discontinuous drug infusion**			
Sodium heparine^i^	0.2 mL	0.2 mL	32.0 ± 2.1; 0.9 ± 0.4
Dopamine chloride		1 000 µg/24 h	24.1 ± 3.4; 0.1 ± 0.1
Vitamine K		1 mg/24 h	17.1 ± 1.1; 0.5 ± 0.2
Caffeine citrate		5 mg/24 h for 30 min	22.7 ± 3.2; 0.8 ± 0.2
Hydrocortisone hemisuccinate		0.5 mg/24 h bolus dose	61.4 ± 1.8; 1.2 ± 0.6
Fluconazole		5 mg/72 h for 1 h	19.3 ± 5.7; 0.3 ± 0.2

^a^Macoflex Macopharma (Mouvaux, France).

^b^Primène Baxter (Maurepas, France).

^c^Viaflo Baxter (Maurepas, France)

^d^Proamp Aguettant (Lyon, France).

^e^Lavoisier (Paris, France).

^f^Phocytan Aguettant (Lyon, France).

^g^Aguettant (Lyon, France).

^h^Levocarnil Sigma-Tau (Issy-les-Moulineaux, France).

^i^Choay Sanofi-Aventis (Paris, France). 5 000 UI/mL sodium heparin was directly added to the parenteral nutrition bags.

^*^According to the USP and EP, the average number of sub-visible particles is <6,000 per container for particle sizes ≥10 µm and <600/container for particle sizes ≥25 µm. Tests showed that all components of the BPN bags present particulate contamination in accordance with the specifications of the Pharmacopoeia. Particulate matter was counted using the APSS-2000 instrument.

#### At the egress of the infusion set by dynamic count (Qicpic instrument)

**Figure 1:**
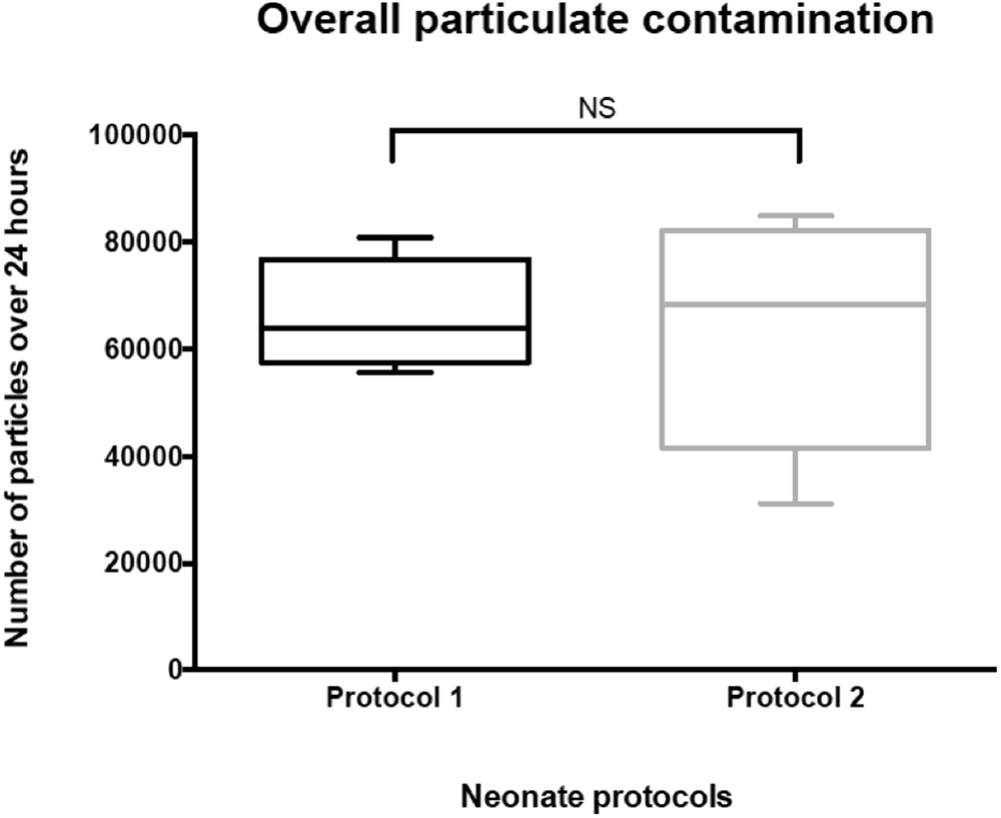
Determination of overall particulate contamination for both neonate protocols infused over a period of 24 hours (N = 5). Results include all particles (spherical and non- spherical).

During infusion, the overall particulate contamination of neonate infusions is expressed as the total number of particles detected by the particle instrument over 24 hours (Fig. 1). No significant difference in total particulate matter was evident from one protocol to the other, with 63,945 [55,562; 80,838] and 68,401 [31,140; 84,855] particles per day for protocols 1 and 2, respectively (P = 0.764). The number of particles ≥10 µm did not differ significantly between the 2 protocols (2,109 [2,911; 5,912] vs. 2,236 [3,977; 6,702] particles per day; P = 0.516). Similarly, no significant differences were observed for the particulate matter of larger particles ≥25 µm, with 18 [6; 51] and 41 [20; 106] particles per day for protocols 1 and 2, respectively (P = 0.146).

#### Between static and dynamic conditions

**Figure 2:**
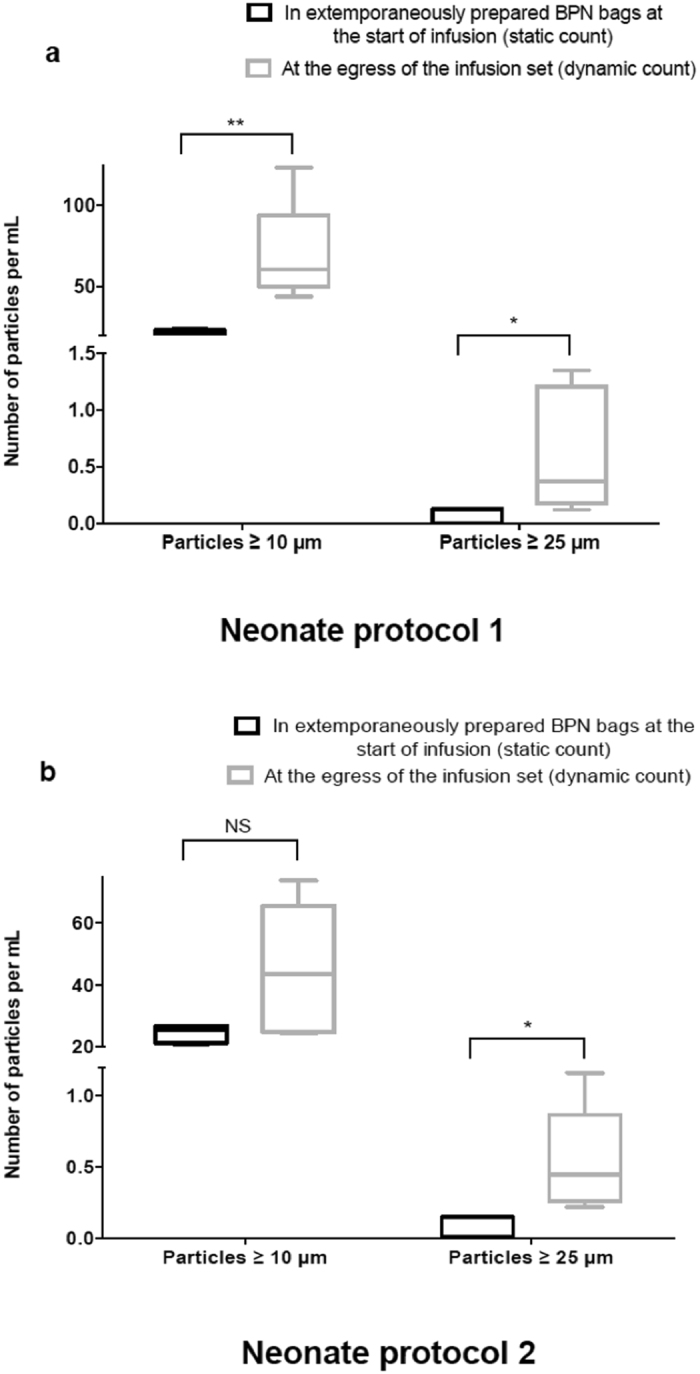
Number of particles in BPN bags at the start of infusion and at the egress of the catheter during a 24-hour infusion for neonatal protocol (**1**) (Fig. 2a) and (**2**) (Fig. 2b). The findings are presented in the form of boxplot, highlighting a significant increase in the number of macroparticles ≥10 µm and 25 µm between the initial particulate matter in the infusion bag and at the egress of the infusion over 24 hours, with either neonate protocol. Only the particulate load ≥10 µm for protocol 2 did not significantly differ, despite an upward trend in the number of particles during infusion (*P* = 0.064). *NS P* > 0.05, *P ≤ 0.05, **P ≤ 0.01.

Results of particle counting are shown in Fig. 2 and are expressed as the median number of particles per mL after taking into consideration the volumes analysed (i.e. 15 mL, 48 mL and 91.2 mL for static and the two dynamic conditions, respectively).

The analysis of particulate matter during neonate protocol infusion demonstrated that the number of particles of ≥10 and 25 µm, increased significantly during infusion, compared to the initial particulate contamination of the BPN bags, for either neonate protocol.

For particles of ≥10 µm, particulate matter increased by at least a factor of 2, for both protocols. Indeed, when infusing protocol 1 (Fig. 2a), particulate matter of ≥10 µm increased from 22.6 [20.6; 23.9] to 60.6 [43.9; 123.2] particles per mL for static and dynamic conditions, respectively (*P* = 0.009). When performing the second protocol, an increase in the number of particles was evident but did not significantly differ, from 25.4 [21.0; 26.8] to 43.6 [24.5; 73.5] particles per mL (*P* = 0.064).

When the analysis focused on particles of ≥25 µm, the infusion of neonate protocol 1 (Fig. 2a) showed a significant increase in macroparticles ≥25 µm (0.0 [0.0; 0.1] vs. 0.2 [0.1; 1.1] particles per mL for static and dynamic conditions, respectively; *P* = 0.034). For neonate protocol 2 (Fig. 2b), the number of particles ≥25 µm significantly increased from 0.0 [0.0; 0.1] to 0.4 [0.2; 1.2] particles per mL for the static and dynamic conditions, respectively (*P* = 0.012).

**Figure 3:**
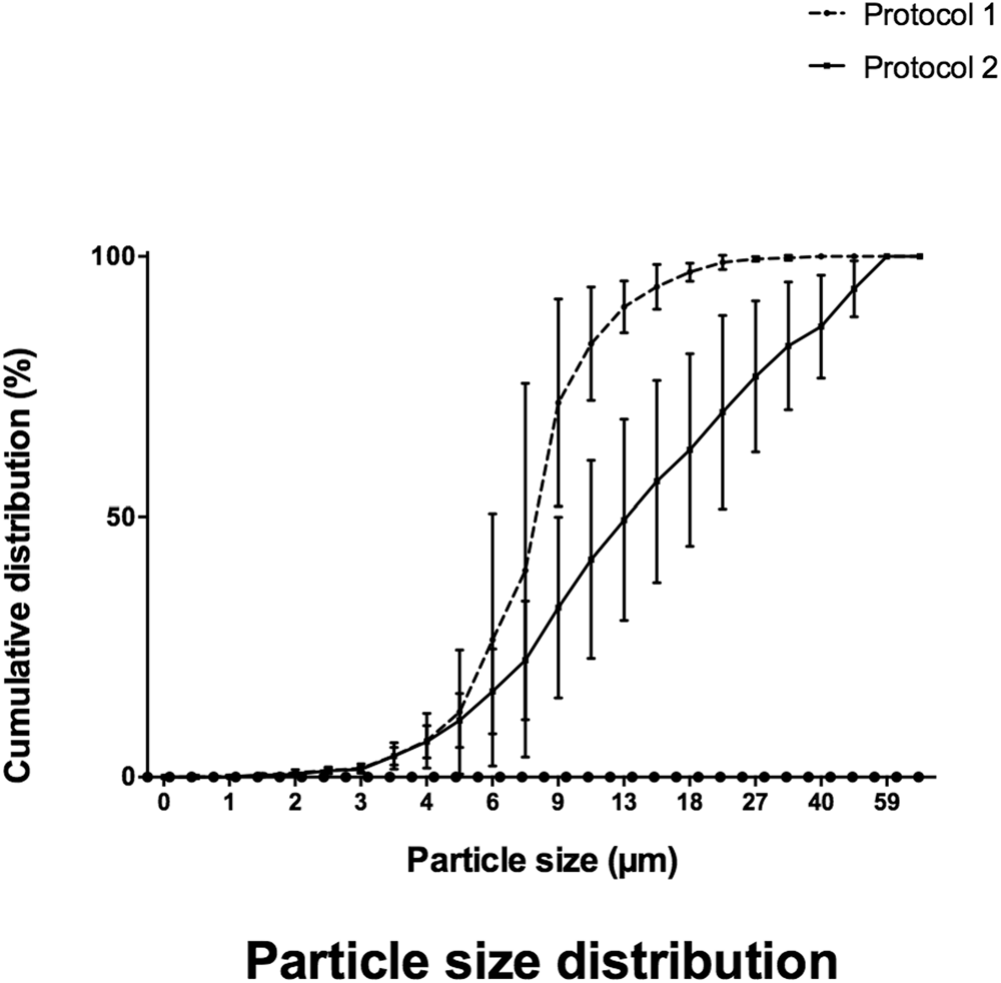
Cumulative distribution of particle sizes for both neonate protocols. Data is presented as means with standard deviation.

During the infusion of either neonate protocol, Fig. 3 demonstrates that, despite very low infusion flow rates, very large particles can potentially be administered to the neonatal population. Thus, whatever the protocol, particles ≥10 µm (up to 50 µm) were infused. Moreover, PSD shows that protocol 2 is characterised by the presence of particles larger than protocol 1, despite reduced particulate matter, especially for macroparticles ≥25 µm.

**Figure 4:**
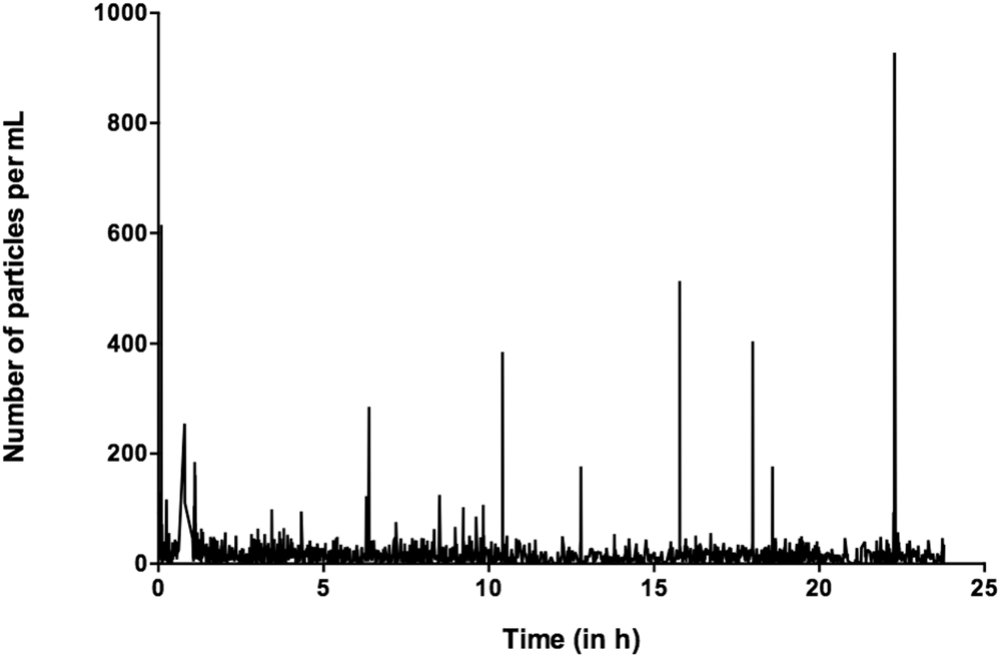
Example of particulate concentration profile over a period of 24 hours (Q(t)) during infusion of protocol (**2**) performed with the Qicpic instrument.

The Qicpic Q(t) module measured the level of particulate contamination over time. An example of Q(t) is shown in Fig. 4 with protocol 2, demonstrating that every IV addition of drugs injected discontinuously was responsible for a significant increase in the particulate matter potentially administered to neonate patients.

## Discussion

The aim of this study was to dynamically quantify particulate matter during NICU practices by simulating multidrug IV therapies. The presence of Luer-lock connectors to the particle-measuring instruments means that the infusion set is directly connected to the system: all particles potentially generated are drawn into the IV tubing to be analysed by the system. Few studies have focused on analysing and quantifying particulate matter during parenteral drug infusions. For the first time, this study has assessed two neonatal protocols commonly used in NICUs by quantifying particulate matter in BPN bags and during their infusion over 24 hours. An important point is to ensure that all drugs administered separately to infants present sub-visible particulate contamination consistent with the Pharmacopoeia^1, 2^. This is also true during their mixing in bags. However, the advantage of our study is to be able to quantify particulate matter when injecting drugs simultaneously during a dynamic IV infusion, demonstrating contamination of the infusion with very large particles.

This result is in accordance with the previous study we performed^18^. Indeed, despite the absence of visible particles in the IV tubing, the particulate analysis showed that patients might receive hundreds of thousands of particles in only one-day multi-drug infusion, an inacceptable situation for very fragile patients. During infusion, two kinds of studies have previously been conducted: 1) indirect quantification of particulate matter with the analysis of membrane in-line IV filters^20^, and 2) impact of particle diffusion in cellular and animal models with a known amount of non-drug particles^7, 20, 21^. All these studies are limited because there is no accurate quantification of the overall particulate matter. However, Ortiz *et al*. quantified sub-visible polyethylene particles during an *in vitro* continuous infusion^22^ and in a previous work, we quantified particulate matter during leukemia treatment in pediatric patients^18^.

Our study is a further step in the evaluation of particulate contamination in therapeutic practice. Particulate matter in injectable drugs is well described by Pharmacopoeias, especially for parenteral drug solutions. Two particle sizes are thus referred to (10- and 25 µm) and have been used to assess the level of particulate contamination of all components in neonate BPN bags using the APSS-2000 system in accordance with the USP and EP. However, Pharmacopoeia methods are not applicable to continuous IV infusion: particles of <10 µm represented more than 99% of the particles counted, which is why another analysing system was used (Qicpic instrument). Our study shows that, even with protocols classically used in ICUs without any visible precipitation, a lot of sub-visible particles are infused to patients (up to 85,000 particles per 24 hours), especially to infants. Furthermore, this study demonstrates that particulate contamination during IV drug infusion is greater than the total particulate matter of all the BPN bag components studied, indicating that the combined infusion of several drugs causes a significant increase in particulate matter. When assessing the particulate matter of particle sizes ≥10 µm and 25 µm, the particle counter showed approximately a two-fold increase, demonstrating the impact of drug incompatibilities during IV infusion. Moreover, the analysis performed during the dynamic infusion of the multidrug protocols showed that particulate matter of ≥10 µm is almost twice the maximal number of sub-visible particles quoted in the Pharmacopoeia. Serious adverse effects of microparticles have previously been reported. Their deleterious effects such as phlebitis or sepsis have often but indirectly been described by comparing with or without the use of in-line filters^23^. Several clinical studies have shown that particles found in infusions might lead to complications, such as respiratory, renal or hematologic dysfunctions, but also inflammation in pediatric patients^8–10^. The brain of the preterm infant is particularly vulnerable to environmental injury. Periventricular leucomalacia and white matter damage are specific brain lesions of the preterm infant associated with poor neurodevelopment. Inflammation and ischemic injury have been recognised as the main cause of perinatal brain injury in the preterm infant. We speculate that particles, in particular the larger (>25 µm) may contribute to brain damage and neurological impairment in preterm babies by inducing inflammation and vascular embolisation.

However, there are limitations to this study. Experiments are required to programme the Qicpic analyser, taking infusion flow rate into account. Low flow rates (~4 mL/h) may cause interindividual variabilities, because of measurement methods. The detection limitation of the particle instrument was set at 1 µm, probably underestimating the neonate’s exposure to particles. During the experiments, lipids were not included in infused drugs. Adding lipids induces the formation of globules that cannot be quantified as solid drug particles. Our *in vitro* results should be interpreted along with clinical data, especially with the conclusions of studies performed in pediatric ICUs. Particulate contamination thresholds defined in previous studies should be reconsidered for neonatology.

In conclusion, our study was performed to develop a new methodology to measure particulate contamination during drug infusion, which is not stated in the Pharmacopoeia. Our method paves the way for *in vitro* assessments comparing infusion practices when several drugs are simultaneously infused through the same venous access, as performed in our NICU. This work indicates that subvisible particles are infused to neonates despite the absence of any visible particles. It highlights the serious problem of particle contamination through IV therapies and concords with international guidelines that recommend the insertion of in-line filters for parenteral nutrition in the preterm baby population^24^. It would be interesting to correlate clinical effects and particulate contamination level.

## Methods

### Instrumentation and Particle Size Analysis

For particle analysis, we used the Qicpic dynamic image analysis instrument (Sympatec GmbH Inc, Clausthal-Zellerfeld, Germany) associated with a Lixell module (Sympatec GmbH). The image acquisition rate is available from 1 to 500 Hz and is synchronised with the high-speed camera that captures images up to 500 frames per second with 1024 × 1024 square pixels. The Qicpic particle analyser with Windox 5.0 software also determines particle sizes of between 1 µm and 30 mm and provides dynamic imaging analysis.

External setups were linked to external connectors of the Lixell module via Luer-Lock connectors. All kinds of stopcocks, valves and extension sets may be used. In our study, the catheter egress of the IV administration set was directly connected to the Qicpic connector, so as to obtain an accurate measurement of the particulate amount administered.

### Experiments – Data Acquisition

**Figure 5:**
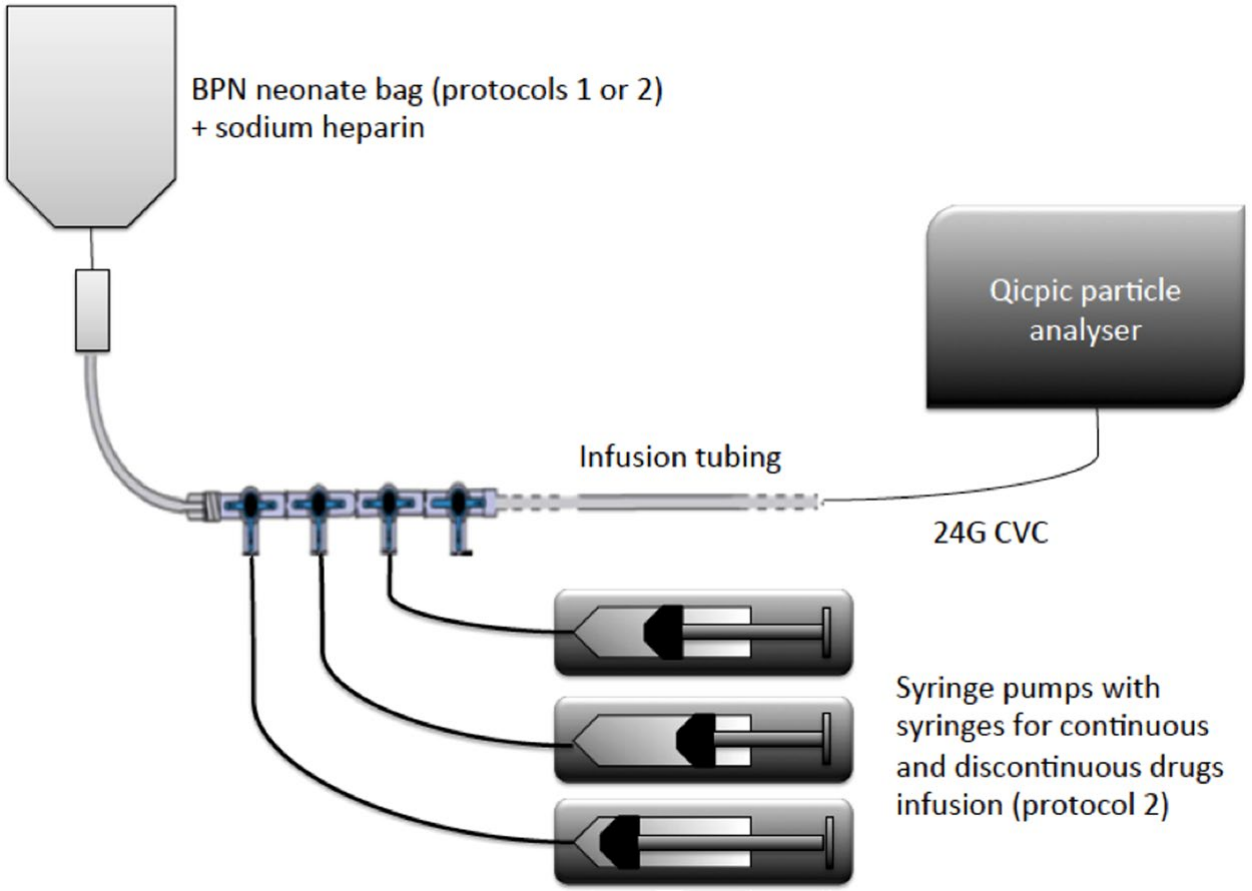
Representation of the two different IV protocols commonly used in our NICU to manage infants.

Two different protocols commonly used in our NICU to manage excessively preterm infants (<1,000 g) were chosen (Fig. 5). Protocol 1 is for the first day after birth in preterm infants requiring only parenteral nutrition. Protocol 2 is for preterm infants with severe cardiorespiratory failure in the first days of life. Protocol 1 consists in a binary parenteral nutrition (BPN) mixture. Protocol 2 associates BPN mixtures with several IV medications, including drugs infused continuously (dopamine) or by slow bolus (caffeine, hydrocortisone, fluconazole). The formulation of BPN bags is shown in Table 1. Only sodium heparin was directly added to both formulations. The infusion of IV therapies was simulated *in vitro*. All BPN bags were infused with a volumetric pump (MVP module Orchestra^®^, Fresenius Vial, Brezins, France) over 24 hours at room temperature. Drugs were infused though syringe pumps (DPS module Orchestra^®^, Fresenius Vial, Brezins, France). The flow rates of BNP bags were 2.0 and 3.8 mL/h for protocols 1 and 2, respectively. For all drugs administered with protocol 2, the total flow rate was 6.4 mL/h. Contrary to normal practice in our NICU, the routine addition of IV in-line filters was omitted and lipids were not included so that our study could determine infused particulate matter accurately.

Due to the very low flow rates applied in the experiments, the lowest frame rate with the Qicpic instrument was required (i.e. 10 Hz). This image acquisition rate is included in all our results.

A 24 G catheter (ref. 218400; Vygon, Ecouen, France) routinely used in the NICU was added at the distal end of each infusion set and connected to the particle counter.

Each protocol was repeated five times over a total period of 24 hours.

Due to the low infusion flow rates used (i.e. 2.0 and 3.8 mL/h), instrument parameters (frame rate, analysis time, number of repeats of the sequence set, programmed breaks) were determined prior to the experiment. For each Qicpic analysis, a tricalcium phosphate suspension, characterised by a homogeneous size of spherical particles, was infused at the same flow rate as in our NICU protocols to determine the delay needed for particles to move from one point to another though the camera (N = 5). This parameter was then used to analyse the particulate matter of our NICU protocols.

### Data presentations

#### Size and shape of particles

The particulate matter of each component used in BPN bag formulation was measured according to USP and EP specifications, using a particle counter (model APSS-2000, PMT, Dourdan, France). Particle sizes were expressed as the diameter of a circle of equal projection area (EQPC) for two sizes (10 µm and 25 µm).

During BPN bag infusion, particle size characteristics were expressed in Feret’s Diameter and Length of Fiber (LEFI), as described in the USP and EP. Feret’s diameter is the distance between imaginary parallel lines tangent to a randomly oriented particle and perpendicular to the ocular scale. LEFI is defined as the longest direct path from one end to another within the particle contour, and is adapted to non-spherical particles. Particle size distribution (PSD) is described as mass-weighted volume distribution.

Shape parameters (sphericity) were used to differentiate air bubbles from particles.

#### Time-dependent contamination of particles

Dynamic particle analysis is associated with a specific Qicpic module, determining the trend in particulate concentration over time.

#### Statistics

All analyses were performed using XLSTAT 3.03 software (Addinsoft, Paris, France). The Student’s t test was used to compare the collected data, after assessing the normality of data distribution by the Shapiro-Wilk test (P-value ≥0.05). For all analyses, statistical significance was indicated by a 2-tailed P-value < 0.05. In the absence of normality in data distribution, statistical significance was indicated by a Mann-Whitney test. Data is expressed as medians [min; max], if not otherwise specified.
